# Ureteroscopy for Urolithiasis in Pregnancy: Outcomes of a Multicentre Study on Behalf of the European Association of Urology Section of Endourology

**DOI:** 10.1016/j.euros.2025.05.003

**Published:** 2025-05-24

**Authors:** Patrick Juliebø-Jones, Vineet Gauhar, Smita De, Øyvind Ulvik, Yiloren Tanidir, Nebil Akdogan, Cemil Aydin, Pablo Contreras, Maria Florencia Frascheri, Javier Pizzarello, Deepak Ragoori, Lazaros Tzelves, Peder Gjengstø, Mathias Sørstrand Æsøy, Albert El Hajj, Elsayed Desouky, Alejandro Bautista-Perez-Gavilan, Vigen Malkhasyan, Olivier Traxer, Christian Beisland, Bhaskar K. Somani

**Affiliations:** a Department of Urology, Haukeland University Hospital, Bergen, Norway; b Department of Clinical Medicine, University of Bergen, Bergen, Norway; c Department of Urology, Ng Teng Fong General Hospital, Singapore; d Department of Urology, Cleveland Clinic Glickman Urological & Kidney Institute, Cleveland, OH, USA; e Department of Urology, Medicana Atasehir Hospital, Istanbul, Turkey; f Department of Urology, Faculty of Medicine, Çukurova University, Adana, Turkey; g Department of Urology, Hitit University School of Medicine, Training and Research Hospital, Corum, Turkey; h Department of Urology, Hospital Alemán de Buenos Aires, Buenos Aires, Argentina; i Department of Urology, University Hospital Southampton NHS Foundation Trust, Southampton, UK; j Department of Urology, Asian Institute of Nephrology and Urology, Hyderabad, India; k Department of Urology, National and Kapodistrian University of Athens, Sismanogleio General Hospital, Athens, Greece; l Department of Urology, American University of Beirut Medical Center, Beirut, Lebanon; m Endourology Academy, Istanbul, Turkey; n Department of Urology, Hôpital Tenon, Paris, France

**Keywords:** Urolithiasis, Pregnancy, Ureteroscopy, Safety

## Abstract

**Background and objective:**

In patients with stone disease during pregnancy, surgical management in the form of ureteroscopy (URS) can be indicated. However, there remains a relatively limited pool of published data. The objective was to perform a multicentre and international study evaluating the safety and outcomes of URS in pregnancy.

**Methods:**

A retrospective study was performed across 12 tertiary endourology centres for patients undergoing URS during pregnancy over the past 10 yr. Data were collected on demographics, diagnostic imaging, operative findings, and intra- and postoperative complications. Follow-up data on stone-free rate (SFR) and pregnancy outcomes were also obtained.

**Key findings and limitations:**

A total of 146 females underwent URS, and the median age was 28 yr (interquartile range [IQR] 24–32). The majority were in the 2nd trimester (58%), were not presented (74%), and had the surgery performed by an endourologist (81%). Ultrasound was the most popular diagnostic imaging modality. Anaesthesia type was as follows: general (62%), spinal (23%), and sedation (15%). The negative URS rate was 48%. The median operative time was 35 min (IQR 35–45), and 77% were discharged within 24 h. The early and late complication rates were 11% and 2.8%, respectively. Regarding the former, there were two intensive care admissions (Clavien IV). In patients in whom a stone was found at URS, the SFR in terms of zero fragments was 65% and it was 78% for residual fragments of ≤2 mm based on postpartum imaging follow-up.

**Conclusions and clinical implications:**

In the setting of a tertiary centre, URS can be performed safely during pregnancy with a low risk of major complications. Future studies are needed to investigate how the diagnostic algorithm can be improved to result in a lower rate of negative URS.

**Patient summary:**

Managing kidney disease in pregnancy is recognised to be challenging as the surgeon must deliver safe care for both the mother and the unborn child. This is even more so the case when surgery to remove a stone is indicated. This study found that performing surgery using a method called ureteroscopy is safe, but it is recommended to be performed in an expert centre.

## Take Home Message

Ureteroscopy can be performed during pregnancy with a complication burden that is not higher than the results published in the literature for index patients. Ultrasound is still considered first choice of imaging modality in pregnant women with acute flank pain, given its availability and lack of associated radiation. Serious complications are uncommon but can occur.

## 1 Introduction

Symptomatic urolithiasis during pregnancy is a relatively uncommon but clinically relevant event [1]. The cumulative incidence of hospital admissions for such a condition has been reported at 1.7 per 1000 deliveries [2]. Potential adverse sequelae associated with urolithiasis in pregnancy include miscarriage, preterm labour, and preeclampsia [3]. The challenge of delivering safe care to both the mother and the unborn child is heightened further by the considerations and restrictions that must be taken regarding pharmacotherapy, ionising radiation, and choice of anaesthesia [4]. Practice patterns vary widely given variability in resources, equipment, and experience of the care team [5]. Surgical intervention can be required, either as part of the diagnostic pathway and/or as a means to definitively treat the stone. Ureteroscopy (URS) is increasingly performed in the index patient, owing to its safety profile and high stone-free rate (SFR), and its use has increased in pregnant patients with urolithiasis accordingly [6]. However, the reports available are largely limited to single-centre series [[7], [8], [9], [10]]. Our goal was to perform an international, multicentre study to investigate the outcomes and morbidity of URS in pregnancy. The secondary aim was to evaluate the diagnostic yield of different imaging modalities, and therefore their reliability, when compared with the final operative findings.

## 2 Patients and methods

A retrospective analysis was performed of pregnant patients (≥18 yr) undergoing URS for suspected stone disease across 12 tertiary endourology centres around the world between January 2014 and December 2023. In addition to the demographic details such as age and trimester, data were collected on diagnostic imaging, operative findings, intra- and postoperative complications, as well as SFR. The latter was categorised as either zero fragments or <2-mm fragments on follow-up imaging, which consisted of either low-dose noncontrast computed tomography or ultrasound (US) depending on local protocols. Bilateral URS surgeries were counted as one procedure only. Early complications (≤30 d) were classified according to the Clavien-Dindo (CD) system. Late complications were for stone-related events >30 d postoperatively and up until the time of delivery.

Approval at each centre’s audit or ethical committee was obtained in accordance with local regulations. Data were anonymised completely before being shared with the coordinating centre. Chi-square test and independent sample *t* test were used to compare categorical and continuous data, respectively. The diagnostic performance of US was assessed by comparing the findings with those at the time of URS. The features assessed included the presence of hydronephrosis and absence of a ureteral jet. Using 2 × 2 contingency tables, we calculated sensitivity, specificity, positive predictive value, and negative predictive value. Multivariable logistic regression was performed to identify the predictors of negative URS among those who had undergone preoperative US.

A statistical analysis was performed using SPSS version 29 (IBM Corporation, Armonk, NY, USA), and significance was determined as *p* < 0.05.

## 3 Results

Overall, 146 pregnant patients underwent URS across 12 centres during the 10-yr study period (Table 1). The majority of these cases (80.8%, *n* = 118) were performed by an endourologist. The median age was 28 yr (interquartile range [IQR] 24–32) and the trimester stages were as follows: first: 12% (*n* = 18), second: 58% (*n* = 85), and third: 30% (*n* = 43). Approximately one in five patients had a pre-existing comorbidity such as hypertension (7.5%), autoimmune disease (3.4%), or diabetes (2.7%). In terms of presenting complaint, flank pain alone was reported in 64% of cases, followed by flank pain with nausea and vomiting in 14%, and nausea and vomiting alone in 11%. The majority of patients (64%, *n* = 93) were not stented earlier.

**Table 1:** Summary of patient demographics and baseline data

Sample size	146
Age (yr), median (IQR)	28 (24–32)
Trimester, % (*n*)	
1st	12 (18)
2nd	58 (85)
3rd	30 (43)
Comorbidities, % (*n*)	19 (28)
Presenting complaint, % (*n*)	
Flank pain	64 (94)
Pain + nausea/vomiting	14 (20)
Nausea/vomiting	11 (11)
Haematuria	3.4 (5)
UTI	2.1 (3)
Flank pain + haematuria	2.1 (3)
Flank pain + UTI	1.4 (2)
Incidental	0.7 (1)
Dysuria	0.7 (1)
Labour pain	0.7 (1)
Preoperative imaging type(s), % (*n*)	
Renal bladder ultrasound	86 (126)
Low-dose CT	4.1 (6)
MRI	3.5 (5)
Plain XR	1.4 (2)
US + MRI	2.1 (3)
US + low-dose CT	2.7 (4)
Findings on preoperative imaging, % (*n*)	
Hydronephrosis	64 (93)
Stone visualised	10 (15)
Absent ureteral jet	26 (38)
Stone size (mm), median (IQR)	8 (6–10)
Preoperative decompression, % (*n*)	
Nil	64 (93)
Nephrostomy	10 (15)
Ureteral stent	26 (38)

CT = computed tomography; IQR = interquartile range; MRI = magnetic resonance imaging; US = ultrasound; UTI = urinary tract infection; XR = x-ray.

### 3.1 Diagnostic imaging and stone findings at the time of operation

Renal bladder US was the most commonly performed imaging modality and was used in 86% (*n* = 126) of cases. No patients underwent transvaginal US. A stone was visualised on preoperative imaging in only 30% (*n* = 44) of cases, with a reported median size of 8 mm (IQR 6–10). The overall negative URS rate was 48% (*n* = 70). Regarding location, 81% (*n* = 67) of the stones identified at the time of surgery were in the ureter.

For patients undergoing preoperative US, sensitivity and specificity for the absence of a ureteral jet were 34% and 80%, respectively. By comparison, for hydronephrosis alone, these values were 85% and 1.5%, respectively. Summary of these findings including when combined are given in Table 2. Among those who had undergone preoperative US, logistic regression revealed that a later trimester was associated with a higher risk of negative URS (odds ratio [OR] 2.2, 95% confidence interval [CI] 1.2–4.3, *p* = 0.001), while older age was associated with a significantly lower odds of negative URS (OR 0.9, 95% CI 0.86–0.98, *p* = 0.002; Supplementary Table 1).

**Table 2:** Summary of diagnostic performance of individual and combined findings on preoperative ultrasound

Ultrasound finding	Sensitivity (%)	Specificity (%)	PPV (%)	NPV (%)
Absence of jet	34	80	61	57
Hydronephrosis	85	1.5	44	41
Absence of jet + hydronephrosis	34	82	63	59

NPV = negative predictive value; PPV = positive predictive value.

### 3.2 Operative results

Anaesthesia choice was as follows: general, 63% (*n* = 91); spinal, 23% (*n* = 33); and sedation only, 5.1% (*n* = 22). The majority of surgeries (63%, *n* = 91) were performed on the right side. The median operative time was 35 min (IQR 25–45; Table 3). Intraoperative US was used in 64% (*n* = 94) of cases, while 7.5% (*n* = 11) used fluoroscopy alone and 27% (*n* = 40) omitted intraoperative imaging completely. The commonest method for stone removal was dusting with laser (68%) followed by the use of a basket only (21%). The exit strategy was as follows: nothing, 5.5% (*n* = 8); standard ureteral stent, 71% (*n* = 104); stent on string, 19% (*n* = 27); ureteral catheter, 2.1% (*n* = 3); and pre-existing nephrostomy left in situ, 2.7% (*n* = 4). Overall, 40% (*n* = 55) of any of these were removed within 14 d of the URS, while the remainder were kept in until the delivery had taken place, with exchanges occurring as per the local protocol. Regarding hospital stay after URS, 66% (*n* = 96) of patients were discharged after a 1 night stay and 10% (*n* = 15) were discharged on the same day as surgery.

**Table 3:** Summary of operative data

Primary surgeon experience, % (*n*)	
Endourologist	81 (118)
Consultant (non–stone specialist)	15 (23)
Resident	3.4 (5)
Anaesthesia type, % (*n*)	
General	62 (91)
Spinal	23 (33)
Sedation only	15 (22)
Operation time (min), median (IQR)	35 (25–45)
Side of surgery, % (*n*)	
Left	34 (50)
Right	62 (91)
Bilateral	3.4 (5)
URS type, % (*n*)	
Semirigid URS only	71 (103)
Flexible URS only	8.2 (12)
Both	21 (31)
Negative ureteroscopy	48 (70)
Location of stone found, % (*n*)	
Ureter	80 (61)
Kidney	20 (15)
Strategy for stone removal, % (*n*)	
Dusting only	68 (58)
Stone basket only	21 (16)
Fragmentation + basket	7.9 (6)
Stone grasper only	2.6 (2)
Successful completion of operation	96.6 (141)
Intraoperative imaging used, % (*n*)	
Ultrasound	64 (94)
Fluoroscopy	7.5 (11)
Ultrasound + fluoroscopy	0.7 (1)
None	27 (40)
Exit strategy, % (*n*)	
Nothing	5.5 (8)
Stent standard	71 (104)
Stent on string	19 (27)
Ureteral catheter	2.1 (3)
Nephrostomy left in situ	2.7 (4)

URS = ureteroscopy.

### 3.3 Complications

The early and late complication rates were 13% (*n* = 19) and 2.1% (*n* = 3), respectively. Regarding the former, urinary tract infection (CD II) was the most common complication (Table 4). Just over half of the early complications were minor in severity (*n* = 10). Six cases (5.1%) required a stent placement (*n* = 1), nephrostomy insertion (*n* = 4), or a repeat URS (*n* = 1) due to persistent pain. No maternal deaths were recorded, but there were two maternal admissions (1.4%) to the intensive care unit (ICU): these were due to a high spinal shock (*n* = 1) after spinal anaesthesia and a septic shock (*n* = 1). The former was the only documented complication that was found to be directly related to anaesthesia. Late adverse events consisted of a urinary tract infection (*n* = 1), acute admission for stent pain (*n* = 1), and repeat URS due to stent encrustation (*n* = 1). One spontaneous abortion was recorded in the early postoperative period in association with maternal admission to the ICU and was possibly related to the stone event. Otherwise, there were no reports of preterm labour after URS.

**Table 4:** Summary of early complications and grade of severity

Type	Management	Clavien grade	% (*n*)
Haematuria	Observation only	I	0.7 (1)
Muscular spasm	Medication	II	0.7 (1)
Pain	Medication	II	1.4 (2)
UTI	Oral antibiotics	II	3.4 (5)
Hypotension (not sepsis related)	Medication	II	0.7 (1)
Pain	Stent insertion	II	0.7 (1)
Pain	Nephrostomy	III	2.7 (4)
Pain	Repeat URS	III	0.7 (1)
Renal dysfunction	Stent	III	0.7 (1)
High spinal shock	ICU admission	IV	0.7 (1)
Septic shock	ICU admission	IV	0.7 (1)

ICU = intensive care unit; URS = ureteroscopy; UTI = urinary tract infection.

Overall, 14% (*n* = 20) of patients experienced some kind of complications after URS. There was no significant difference in the overall complication rate according to the trimester (1st: 17%, 2nd: 11%, and 3rd: 19%; *p* = 0.4). There was also no significant difference in complication rates depending on whether a stone was found at the time of surgery or not (12% vs 16%, *p* = 0.4).

### 3.4 Stone-free rate

At follow-up imaging, the zero-fragment SFR was 65%, and for residual fragments of ≤2 mm, it was 78%.

## 4 Discussion

The overall postoperative complication rate for URS among index patients has been reported between 4% and 25% [11]. These are mostly minor in nature, but postoperative sepsis rates up to 5% are reported according to the European Association of Urology (EAU) guidelines [11]. The overall rate of 14% in our study therefore sits within these figures. URS in pregnancy is still a controversial topic. This is particularly with regard to whether it should serve a diagnostic role or it should be reserved as only a therapeutic intervention when a stone has been confirmed on preoperative imaging. Whether computed tomography (CT) should be implemented more frequently on a routine basis in the diagnostic pathway for suspected urolithiasis in pregnancy heightens disagreement further. EAU guidelines give CT as a last-line option that should be employed judiciously [11]. The American Urological Association guidelines do not make a specific recommendation but refer to the American College of Obstetricians and Gynecologists recommendations that allow for its use when deemed medically necessary [[12], [13], [14], [15]]. Given that nearly one in two patients in this study had no stone identified at URS, it prompts the question as to whether preoperative imaging in the form of CT or magnetic resonance imaging (MRI) should serve a larger role in the diagnostic workup and whether the risks are potentially lower than those associated with a surgery that reveals negative findings. The results from the diagnostic performance of US features reveal that the absence of a ureteral jet is a stronger predictor of a stone being present than hydronephrosis, which had very low specificity. Given the known phenomenon of physiological hydronephrosis during pregnancy, especially on the right side, it is a poor standalone finding to guide management. In clinical terms, the specificity of 82% means that if a patient has hydronephrosis *as well as* no ureteral jet, the chance of finding a stone at URS is quite high.

SFRs reported in URS series in pregnant patients should be interpreted with caution and are difficult to compare with those in the general population. Firstly, the same imaging modality is seldom used pre- and postoperatively. Secondly, treatment with URS is largely aimed at treating the culprit stone, rather than targeting the entire stone burden. At the same time, if the latter is not large and if it is considered safe at the time of surgery to do so, achieving full stone clearance could offer added benefits. While SFR is commonly used as a marker of success among index patients, in the setting of pregnant patients undergoing URS, success should arguably be considered in terms of relieving the acute episode for the mother to enable her to go on and deliver the child safely.

Avoiding URS and opting for a temporising measure such as a ureteral stent or percutaneous nephrostomy (PCN) for the remainder of the pregnancy is a viable alternative and one that is often adopted, especially outside the setting of a tertiary endourology centre. However, not only encrustations and the need for frequent exchanges can be very burdensome for the patient, but also the total radiation exposure can be higher [16]. Close follow-up is therefore needed in patients in whom either one of these has been placed. Scheduled exchanges with follow-up every 3 wk have been proposed to avoid potential complications [17]. In a study by Lyon et al [18], the total mean radiation exposure was 286.9 mGy for PCN versus 0.2 mGy for primary URS (*p* < 0.001). The average time between PCN exchanges in this particular study was 16.5 d. The same centre performed another study, which found that anaesthesia type and timing for stone procedures during birth were not associated with preterm birth [19].

Our study is limited by its retrospective status, and it should be noted that the data were collected from tertiary endourology centres only. Data were also lacking on preoperative antibiotic use, venous thromboembolism prophylaxis, and laser type used. None of the participating centres routinely evaluate renal Resistive Index, which has previously been found to be a useful diagnostic adjunct [20]. In addition, the low number of CT and MRI scans performed preoperatively precluded an analysis of their diagnostic performance given the high potential for overinterpretation of random variations. A lack of preoperative CT also negates obtaining stone volume in patients.

Despite these shortcomings, this study represents one of the largest studies of its kind and, to our knowledge, one with the highest number of centres involved worldwide. The low incidence of URS in pregnancy represents a challenge when performing prospective studies. Furthermore, the inherent clinical equipoise associated with management of stones in pregnancy arguably renders it unfeasible at this time to initiate randomised studies.

## 5 Conclusions

In the setting of a tertiary centre, URS can be performed during pregnancy with a low risk of major complications. Future studies are needed to investigate how the diagnostic algorithm can be improved to reduce the rate of negative URS.

  ***Author contributions*:** Patrick Juliebø-Jones had full access to all the data in the study and takes responsibility for the integrity of the data and the accuracy of the data analysis.

  *Study concept and design*: Juliebø-Jones, Gauhar, Somani.

*Acquisition of data*: Juliebø-Jones, Gauhar, De, Ulvik, Tanidir, Akdogan, Aydin, Contreras, Frascheri, Pizzarello, Ragoori, Tzelves, Gjengstø, Æsøy, El Hajj, Desouky, Gavilan, Malkhasyan, Traxer, Beisland, Somani.

*Analysis and interpretation of data*: Juliebø-Jones, Gauhar, De, Ulvik, Beisland, Somani.

*Drafting of the manuscript*: Juliebø-Jones, Gauhar, Somani.

*Critical revision of the manuscript for important intellectual content*: Juliebø-Jones, Gauhar, De, Ulvik, Tanidir, Akdogan, Aydin, Contreras, Frascheri, Pizzarello, Ragoori, Tzelves, Gjengstø, Æsøy, El Hajj, Desouky, Gavilan, Malkhasyan, Traxer, Beisland, Somani.

*Statistical analysis*: Juliebø-Jones, Somani.

*Obtaining funding*: None.

*Administrative, technical, or material support*: None.

*Supervision*: Somani, Traxer.

*Other*: None.

  ***Financial disclosures:*** Patrick Juliebø-Jones certifies that all conflicts of interest, including specific financial interests and relationships and affiliations relevant to the subject matter or materials discussed in the manuscript (eg, employment/affiliation, grants or funding, consultancies, honoraria, stock ownership or options, expert testimony, royalties, or patents filed, received, or pending), are the following: None.

  ***Funding/Support and role of the sponsor*:** None.

  ***Ethics statement*:** Approval at each centre’s audit or ethical committee was obtained in accordance with local regulations.
