# Supplementary material for: Ureteroscopy for Urolithiasis in Pregnancy: Outcomes of a Multicentre Study on Behalf of the European Association of Urology Section of Endourology
Source: Eur Urol Open Sci. 2025 May 24;76:65–70. doi: 10.1016/j.euros.2025.05.003 (PMC12159928; doi:10.1016/j.euros.2025.05.003)
Supplement: Supplementary Data 1 [file mmc1.docx]

|  | **Multivariable** | | |
| --- | --- | --- | --- |
| **Characteristic** | **OR**^1^ | **95% CI**^1^ | **p-value** |
| **Trimester** | 2.2 | 0.92, 1.1 | 0.02 |
| **Age** | 0.92 | 0.86, 0.98 | 0.01 |
| **Comorbidity**  No  Yes | 0.79 | Reference  0.28, 2.2 | 0.7 |
| **Side of surgery**  Right  Left | 1.2 | Reference  0.55, 2.8 | 0.6 |
|  |  |  |  |

Supplementary Table 1. Multivariable logistic regression among patients with pre-operative ultrasound only, to identify predictors for negative ureteroscopy.

OR = Odds ratio

CI = Confidence interval
